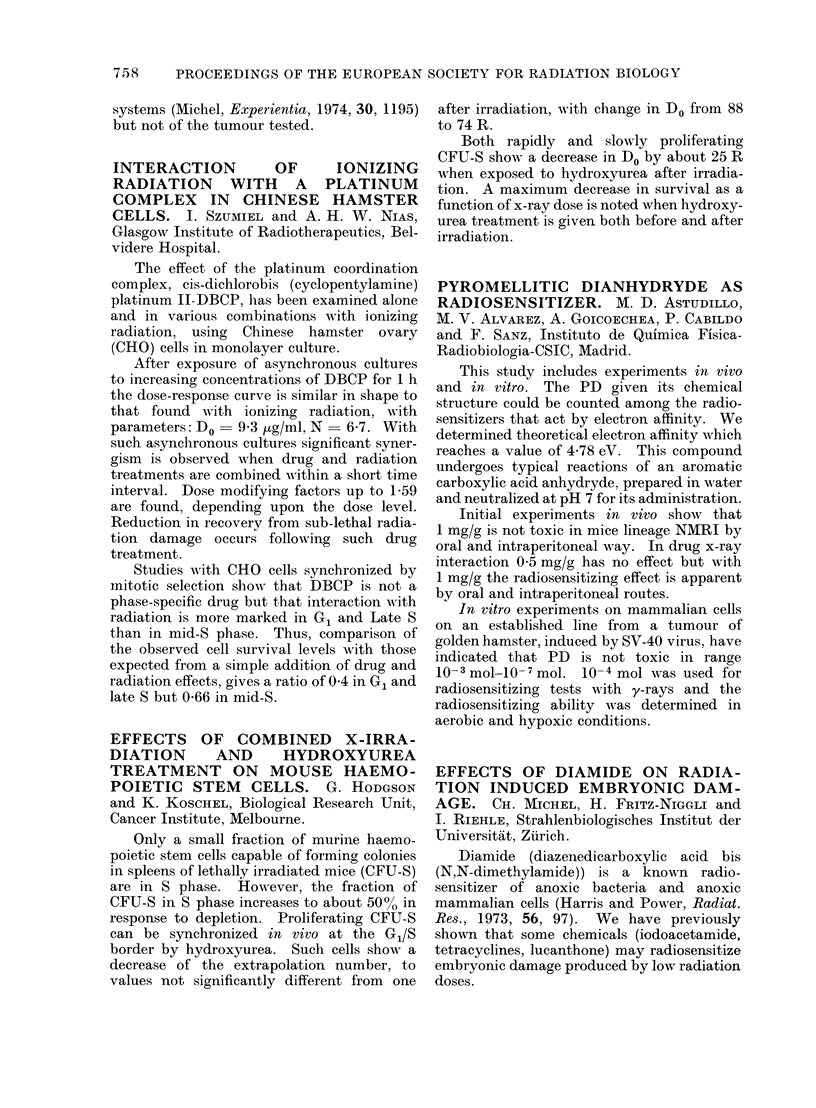# Proceedings: Interaction of ionizing radiation with a platinum complex in Chinese hamster cells.

**DOI:** 10.1038/bjc.1975.311

**Published:** 1975-12

**Authors:** I. Szumiel, A. H. Nias


**INTERACTION OF IONIZING RADIATION WITH A PLATINUM COMPLEX IN CHINESE HAMSTER CELLS.** I. SZUMIEL and A. H. W. NIAS, Glasgow Institute of Radiotherapeutics, Belvidere Hospital.

The effect of the platinum coordination complex, cis-dichlorobis (cyclopentylamine) platinum II-DBCP, has been examined alone and in various combinations with ionizing radiation, using Chinese hamster ovary (CHO) cells in monolayer culture.

After exposure of asynchronous cultures to increasing concentrations of DBCP for 1 h the dose-response curve is similar in shape to that found with ionizing radiation, with parameters: $D_0 = 9{\cdot}3\ \mu g/ml$, $N = 6{\cdot}7$. With such asynchronous cultures significant synergism is observed when drug and radiation treatments are combined within a short time interval. Dose modifying factors up to 1·59 are found, depending upon the dose level. Reduction in recovery from sub-lethal radiation damage occurs following such drug treatment.

Studies with CHO cells synchronized by mitotic selection show that DBCP is not a phase-specific drug but that interaction with radiation is more marked in $G_1$ and Late S than in mid-S phase. Thus, comparison of the observed cell survival levels with those expected from a simple addition of drug and radiation effects, gives a ratio of 0·4 in $G_1$ and late S but 0·66 in mid-S.